# The Eucalyptus in Malarial Disorders

**Published:** 1878-12

**Authors:** 


THE EUCALYPTUS IN MALARIAL DISORDERS.—Dr. H. B. Dow, of London, contributes the following experience with this remedy to the London *Lancet:*

In the first case in which I tried it, it was suggested to me by my patient, he saying that he had taken quinine by the pound without result, and that the eucalyptus was the only remedy for him. He had many years since contracted malaria of the worst type in the Douro district, and had tried most remedies without avail. A very few doses of eucalyptus globulus removed the symptoms.

In the second case, my patient had been many years abroad as a missionary, and suffered severely from intermittent fever contracted during his labors in tropical climates. He also found no relief from quinine, but was very speedily relieved by the eucalyptus.

In the third case, my patient was a gentleman who had lived many years in India and China, and during his residence abroad had had even attacks of ague. Recently he experienced a return of his old symptoms, and took quinine, as he had been accustomed to, to check the illness. However, on this occasion it failed to produce the usual effect, so I recommended him to try the eucalyptus. The effect was at once marked, and speedily all his intermittent symptoms left him

